# Neuroendoscopic Transventricular Approach for Cystic Craniopharyngioma

**DOI:** 10.7759/cureus.18123

**Published:** 2021-09-20

**Authors:** Mohammad Hassan A Noureldine, Sajjad Khodmehr, Mohammadmahdi Sabahi, Puya Alikhani, George I Jallo, Mahdi Arjipour

**Affiliations:** 1 Neurosurgery and Brain Repair, University of South Florida Morsani College of Medicine, Tampa, USA; 2 Neurosurgery Research Group (NRG) Student Research Committee, Hamadan University of Medical Sciences, Hamadan, IRN; 3 Neurological Surgery, Neurosurgery Research Group (NRG) Student Research Committee, Hamadan University of Medical Sciences, Hamadan, IRN; 4 Neurosurgery and Brain Repair, University of South Florida, Tampa, USA; 5 Neurosurgery, Johns Hopkins All Children's Hospital, Baltimore, USA; 6 Neurosurgery, Brain and Spinal Cord Injury Research Center, Neuroscience Institute, Tehran University of Medical Sciences, Tehran, IRN; 7 Neurosurgery, School of Medicine, Hamadan University of Medical Sciences, Hamadan, IRN

**Keywords:** ommaya reservoir, microsurgery, neuroendoscopy, cystic, craniopharyngioma

## Abstract

The literature is rich with many studies reporting different treatment modalities and approaches for cystic craniopharyngioma (CC), including microsurgery, neuroendoscopic transventricular approach, endoscopic transnasal surgery, stereotactic drainage, and Ommaya reservoir insertion. The goals of this manuscript are to report the successful treatment of an atypical case of CC using the neuroendoscopic transventricular approach (NTVA) as well as discuss the different surgical modalities for these tumors following a comprehensive review of the literature. Our patient is a nine-year-old female with a large CC who was managed using the NTVA. No complications or recurrence occurred over two years of follow-up. Results of our literature review showed lower recurrence and complication rates of the NTVA compared to other surgical modalities.The NTVA is potentially efficient, reliable, and safe for managing CC and cystic-dominant craniopharyngiomas, with low recurrence and complication rates compared to microsurgery and Ommaya reservoir insertion. Future randomized clinical studies comparing the various treatment modalities of CC are needed to solidify these conclusions.

## Introduction

Craniopharyngiomas arise from squamous cells of Rathke’s pouch. These benign tumors account for 10% of pediatric and 2-4% of intracranial brain tumors, are located in the intra- and suprasellar zones, and are classified as cystic, solid, or mixed [1]. Tumoral attachment to critical neurovascular tissue, such as the hypothalamus or pituitary, is a treatment challenge. Although endocrine dysfunction could be seen in total resections, the risk is increased in partial resections as well [2]. Total or partial surgical resection remains the treatment of choice, and radiation therapy may play a role in partial resections or recurrences. Neuroendoscopic transventricular fenestration and placement of an intracystic catheter with an Ommaya reservoir is a valid, minimally invasive technique in the management of large cystic craniopharyngiomas (CC) [3-4]. The objectives of this paper are to report the management of an atypical CC using the neuroendoscopic transventricular approach (NTVA), as well as discuss the different surgical modalities for these tumors following a review of the literature.

## Case presentation

**Figure 1:**
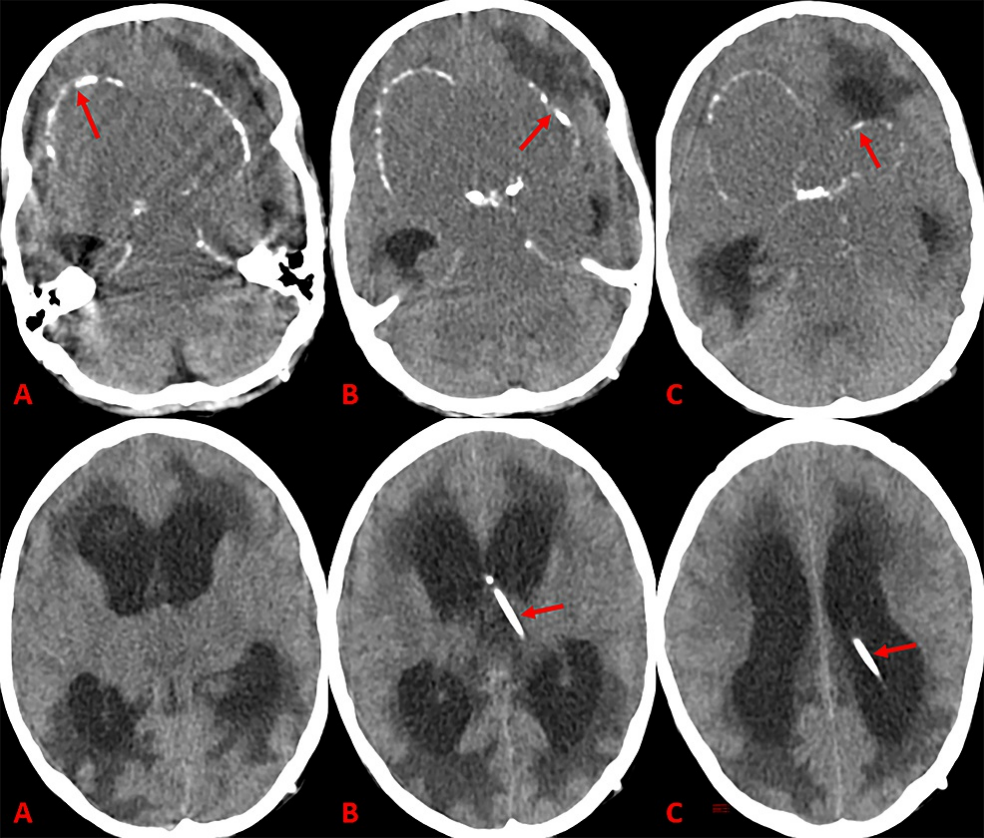
Head CT scan images reveal a massive cystic lesion with extension from the anterior to posterior fossa and upward to the lateral ventricles Cystic contents are iso-to-hypodense and peripheral punctate calcifications (arrows) are evident (A-C). Diffuse periventricular hypodensity due to interstitial edema (D, arrow) initially persisted despite shunt placement (E & F, arrows).

**Figure 2:**
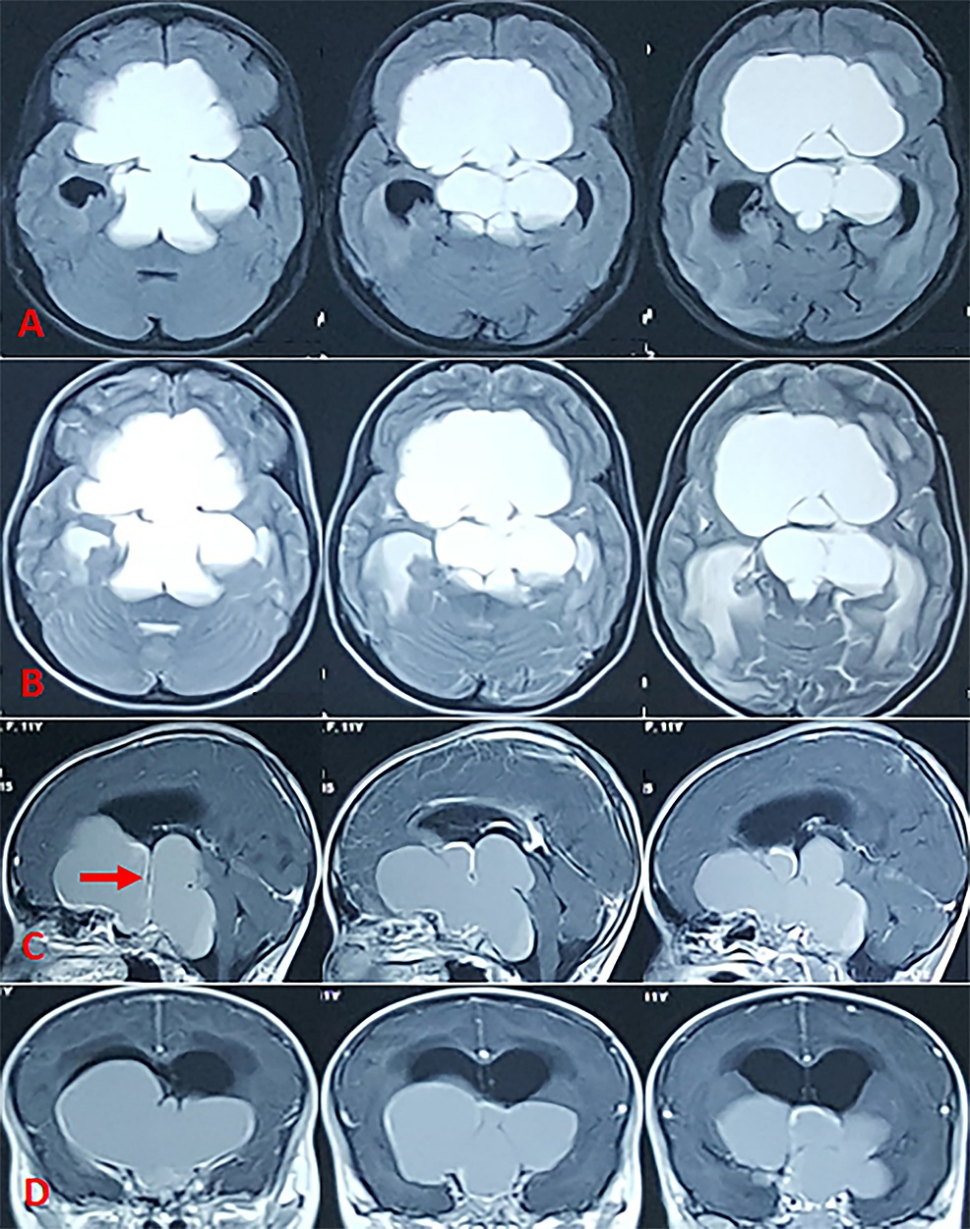
MRI of the brain Brain MRI showing a diffuse hyperintense signal on axial fluid-attenuated inversion recovery (FLAIR) (A) and axial T2-weighted images (B), which is consistent with high levels of protein and cholesterol. Sagittal (C) and coronal (D) sections of postcontrast T1-weighted images reveal no significant enhancement of the solid component. All these findings support the diagnosis of cystic craniopharyngioma. The lesion extended from anterior to posterior fossa and laterally to the middle fossa, compressing and elevating all basal elements (frontal lobes, temporal lobes, third ventricle, brain stem). The chronically compressed pituitary infundibulum has been elongated and pushed superiorly as shown (arrow in C).

The patient is a nine-year-old female who presented with headache, agitation, urinary frequency, and progressive visual deficits over the past two years. A large sellar and suprasellar lesion was detected on a CT scan, and a ventriculoperitoneal (VP) shunt was placed to relieve the progressive symptoms of hydrocephalus (Figure 1). MRI solidified the initial suspicion of CC, where the lesion was hyperintense on T1- and T2-weighted images, without any significant, enhancing solid component on postcontrast T1-weighted images, consistent with the high protein and cholesterol contents of CC (Figure 2).

A few days prior to surgery and despite no signs of VP shunt failure, the patient’s exam was notable for acute confusion, severe ataxia, and bilateral blindness. The NTVA was performed using a rigid neuroendoscope (Aesculap AG, Tuttlingen, Germany). The cyst was accessed through a right frontal burr hole, and the contents were drained. The neuroendoscope was advanced to the right lateral ventricle, where cyst wall calcifications were visible through the thinned and elevated neural elements. The choroid plexus was then identified and tracked back to the foramen of Monro. The cyst wall was sampled and the cyst was punctured, draining a copious gray-to-green liquid content with debris. Simultaneous irrigation with a Ringer’s solution and suctioning cleared the contents of the cyst; adjacent neurovascular structures were clearly visible at the end of the procedure.

**Figure 3:**
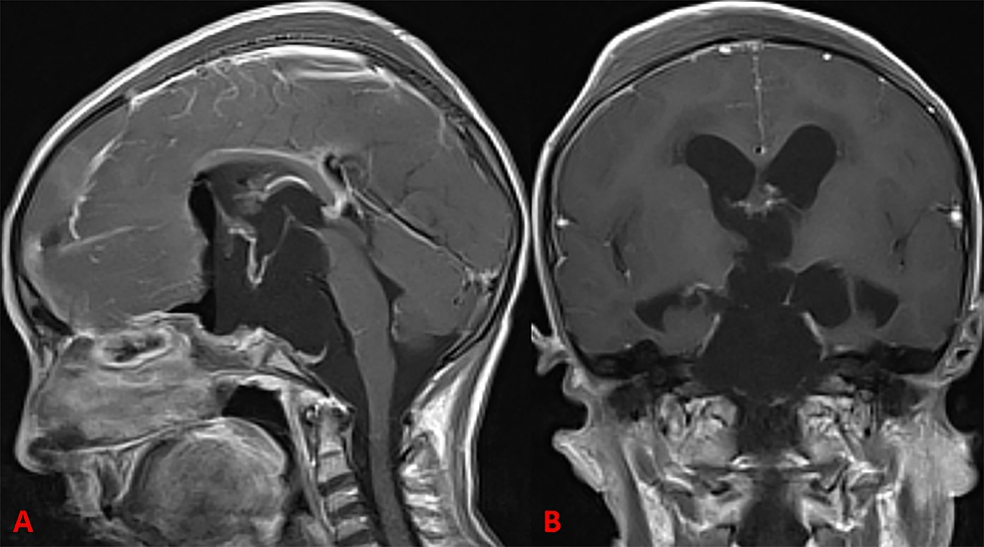
Immediate postoperative MRI Sagittal (A) and coronal (B) postcontrast T1-weighted images showing complete evacuation of cystic contents, decompression of neurovascular elements, and no obvious solid tumor component.

**Figure 4:**
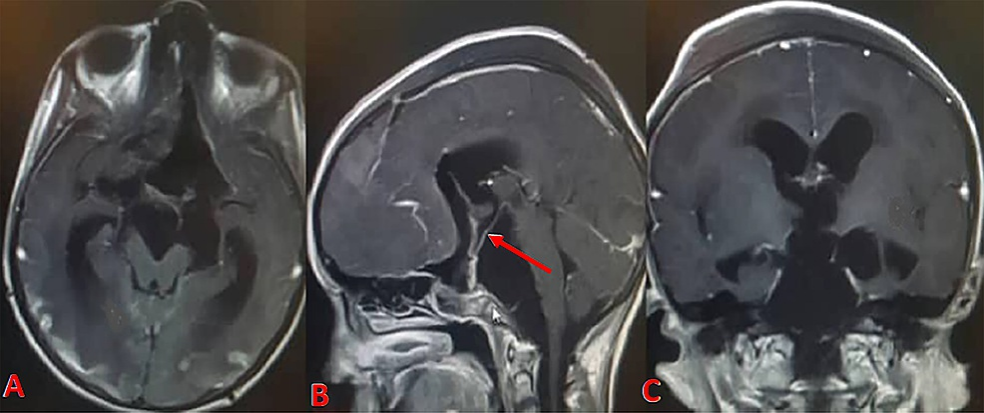
Follow-up MRI at two years Axial (A), sagittal (B), and coronal (C) postcontrast T1-weighted images revealing no re-accumulation of cystic contents. The elongated infundibulum of the pituitary gland is noted (arrow in B).

The postoperative course was uneventful, and all symptoms improved in the immediate postoperative period, except for visual loss. Histopathology confirmed the diagnosis of CC. The patient did not develop any symptoms of chemical meningitis on close follow-up. Her pituitary function remained normal postoperatively as well as on long-term follow-up. We opted not to pursue any additional surgical or adjuvant therapy due to the lack of any obvious solid tumor component on postoperative MRI, very thin residual cyst wall, and extensive basal extension with involvement of the critical structures (Figure 3). Follow-up MRIs showed no evidence of tumor regrowth or re-accumulation of cystic contents (Figure 4).

## Discussion

In this paper, we report a child with a large CC, which was treated via NTVA only with no complications or recurrence after two years of follow-up. As there is a lack of consensus regarding the optimal surgical approach to manage CC, we conducted a literature review about the different treatment modalities and summarized our results in tables.

We found 17 reports that utilized the NTVA (with and without Ommaya reservoir insertion) for the treatment of CC in 67 patients, with a follow-up period ranging from six to 73 months; the recurrence rate ranged between nil to 54% (Table 1) [2-3,5-19].

**Table 1:** Summary of the literature reporting the neuroendoscopic transventricular approach in the management of cystic craniopharyngioma CSF, cerebrospinal fluid; mo., month(s); N, number of patients; NA, not available; NR, not reported; Pt(s): patient(s); yrs., years

Author(s) (year)	N	Age (yrs.)	Characteristics	Location	Signs & Symptoms	Surgical drainage	Radiotherapy	Follow-up (mo.)	Recurrence	Comments
Hellwig et al. (1995) [5]	5	NR	Cystic; mixed	NR	NR	Endoscopy (CSF)	No	NR	No cyst recurrence	40% reported for the solid portion
Nakamizo et al. (2001) [6]	1	3	Cystic	Third ventricle	Gait disturbance; somnolence	Endoscopy (CSF)	No	24	No	Nearly disappearing cystic tumor; no clinical meningitis or neurologic deficit
Joki et al. (2002) [7]	1	10	Cystic	Sella, pons, & medulla oblongata	Headache; visual acuity impairment	Endoscopy (CSF)	Yes	6	No	Complete cystic decompression; visual acuity and headache improvement
Delitala et al. (2004) [8]	7	9–72	Cystic; recurrence (43%)	Supradiaphragmatic; parachiasmatic; extraventricular (4 Pts); intra- + extraventricular (1 Pt); purely intraventricular (2 Pts)	NR	Endoscopy (CSF)	14% (pre)	38	28%	No chemical meningitis
Nakahara et al. (2004) [3]	3	46–76	Cystic	Suprasellar	Headache; dementia; incontinence; hemianopsia	Endoscopy (CSF)	Yes	7	33%	Decompression of the optic chiasm
Tirakotai et al. (2004) [9]	10	NR	Mixed	NR	NR	Endoscopy (CSF)	No	NR	No cyst recurrences; 20% re-operated for solid portion	None
Kamikawa & Inui (2005) [10]	1	4	Cystic	Suprasellar	Visual disturbance; headache	Endoscopy (CSF)	Yes	NR	NR	No chemical ventriculitis or meningitis
Berlis et al. (2006) [11]	1	63	Cystic; recurrence	NR	Focal neurological deficits	Endoscopy (CSF)	No	6	No	Evident decrease of cyst size
Cinalli et al. (2006) [12]	1	NA	Cystic	NA	NA	Endoscopy (CSF)	No	12	No	None
Fujimoto et al. (2007) [13]	1	74	Mixed	Suprasellar region, extending cranially & obstructing the foramina of Monro bilaterally	Headache; dizziness & gait disturbance	Endoscopy (CSF)	No	48	No	Symptoms improved, except for hemianopsia; reduction of cyst size
Cappabianca et al. (2008) [14]	1	3	Cystic	Intraventricular	Headache; vomiting; bilateral papilledema	Endoscopy (CSF)	No	36	No	None
Park et al. (2011) [15]	13	26	Cystic	NR	NR	Endoscopy	Yes	32	54%	Ommaya reservoir kept in place postoperatively; visual fields stable or improved in 12 Pts (92.3%); preservation of endocrine function
Mohanty et al. (2013) [16]	3	44.6 (18–63)	Solid (2 Pts); cystic (1 Pt)	Intraventricular	Headache; visual disturbance; drowsiness; memory impairment; confusion	Endoscopy	Yes (1 Pt)	11.6	No	Weakness & memory impairment improved
Takano et al. (2015) [17]	9	56.7 (35–88)	Cystic	Sellar & suprasellar (2 Pts) Suprasellar (7 Pts)	Raised intracranial pressure; headache; memory disturbance; visual disturbance; hypopituitarism	Endoscopy (CSF)	Yes	73	11%	Tumor size reduction; symptoms improved
Shukla (2015) [18]	3	5–12	Cystic	Small calcified suprasellar tumor; large cyst extending into the third ventricle	Raised intracranial pressure	Endoscopy (CSF)	Yes	6–11	No	Collapse of the cyst; subsidence of hydrocephalus
Moore et al. (2017) [19]	2	7	Cystic	Suprasellar	Headache; nausea; vomiting; somnolence; behavioral changes; decreased visual fields	NR	No	24	No	None
Lauretti et al. (2018) [2]	8	43 (32–52)	Cystic; mixed	NR	Raised intracranial pressure; hypothalamic or pituitary dysfunction; visual disturbances	Endoscopy (CSF)	12.5% (pre)	56	12.5%	None

The surgical modality of choice may differ depending on patient- and/or tumor-related factors. In pediatric and adult patients with good functional status, long life expectancy, and small tumor size, microsurgical gross-total resection (GTR) is the recommended strategy [3]. GTR may be considered an acceptable option for tumors not invading the hypothalamus due to the low risk of recurrence and to avoid subsequent radiotherapy. Tumors invading the pituitary stalk, however, are potentially better managed with sub-total resection (STR) due to the high risk of postoperative endocrinopathy [18]. Tumor recurrence, reaching up to 62% (Table 2), is the main concern following microsurgery, endoscopic endonasal, and/or Ommaya reservoir insertion [15,17,20-60]. Alternatively, see Table 2 for the recurrence rate of CC managed with NTVA (without Ommaya reservoir insertion) ranged between 0% and 33% across various studies.

**Table 2:** Summary of the literature reporting microsurgical, endoscopic endonasal, and Ommaya reservoir procedures for patients with cystic craniopharyngioma GTR, gross-total resection; mo., month(s); N, number of patients; NA, not available; NR, not reported; NTR, near-total resection; PR, partial resection; Pt(s), patient(s); STR, sub-total resection; yrs., years

Author(s) (year)	Surgical Approach	Approach Details	N	Age (yrs.)	Characteristics	Postoperative Visual Function	Follow-up (mo.)	Recurrence
Yasargil (1996) [20]	Microsurgery	NA	162	NA	NA	NA	NA	10%
Fahlbusch et al. (1999) [21]	Microsurgery	Among 148 primary Pts: GTR 73, STR 33, PR 21, Biopsy 4, No resection 17 Among 34 Pts with recurrence: GTR 12, STR 7, PR 15	Primary 148 (Adults 118 – Children 30); Secondary 20	Primary surgery: 36.6 (1 – 79)	Among 148 primary Pts: Purely cystic 5, Predominantly cystic 73, Predominantly solid 42, Purely solid 16, Multicystic 12; Among 34 Pts with recurrence: Purely cystic 1, Predominantly cystic 15, Predominantly solid 12, Purely solid 2, Multicystic 4	Primary Surgery: Normalized 38%, Improved 35%, Unchanged 14%, Worsened 12%; Surgery for recurrence: Normalized 12% Improved 48% Unchanged 32% Worsened 8%	GTR (30 Pts) 120; STR (86 Pts) 60; Mean 64.8	GTR 19%; STR 52%
Van Effenterre & Boch (2002) [24]	Microsurgery	GTR 72; STR 35; PR 15	122	32.7 (1.5 – 78)	NR	Of 76 Pts followed: Normalized 34%; Improved 48%; Unchanged 3%; Worsened 14%	Mean 84	24%
Im et al. (2003) [22]	Microsurgery	GTR	6	10.6	Cystic	All improved	Mean 23	16%
Karavitaki et al. (2005) [23]	Microsurgery	GTR 16 (Group A), GTR + Radiotherapy 3 (Group B), PR 51 (Group C), PR + Radiotherapy 33 (Group D), Cyst evacuation 6 (Group E), Cyst evacuation + Radiotherapy 3 (Group F)	121	Age <16: 42, Age ≥16: 79, Total: 2.5 – 83	Purely or predominantly cystic 42/91, Mixed 33/91, Purely or predominantly solid 16/91	Worsening of visual fields at 10 yrs.: Group A: 9%, Group B: 0%, Group C: 45%, Group D: 24%	Mean 103	GTR 0%, PR 62%
Filis et al. (2009) [31]	Microscopic-endoscopic	GTR	1	7	Cystic	NR	24	No
Schubert et al. (2009) [38]	Microsurgery	GTR 6, SR 11	17	≤17	Cystic	NR	66	58%
Ichikawa et al. (2016) [34]	Microscopic-endoscopic	GTR	4	6.4	Cystic	Improved 2, Unchanged 2	Mean 142	25%
Feng et al. (2018) [30]	Microsurgery	GTR 124, STR 37, PR 13	183	36.2 (3 – 77)	NR	Improved 54, Worsened 22	27.3	12%
Shibata et al. (2018) [39]	Microsurgery + Endoscopic endonasal	GTR	1	1	Cystic	Improved (light perception)	18	No
Abe et al. (1997) [25]	Endoscopic endonasal	GTR 15 STR 19	35	27 (8 – 72)	Cystic	Improved 18 Unchanged NR Worsened NR	Mean 24.1	8.6%
Buhl et al. (2001) [26]	Endoscopic endonasal	NR	1	4	Cystic	Improved	12	No
Fujimoto et al. (2002) [32]	Endoscopic endonasal + Radiation therapy	GTR	1	8	Cystic	Improved	30	No
Locatelli et al. (2004) [36]	Endoscopic endonasal	GTR 1, Draining cyst contents to sphenoid sinus 3, Multiphase approach 1	5	2 – 16	Cystic	NR	Mean 48	20%
Chakrabarti et al. (2005) [28]	Endoscopic endonasal	GTR 61, PR 7	68	2.5 – 73	Cystic & solid	Improved 54, Unchanged 12, Worsened 2	Mean ≥66	10%
Rudnick & DiNardo (2006) [37]	Endoscopic endonasal	-	1	31	Cystic	NR	28	No
Gardner et al. (2008) [33]	Endoscopic endonasal	GTR 11, PR 5	16	55 (36 – 80)	NR	Improved 13, Unchanged 1, Worsened 0	Mean 34	11%
Stamm et al. (2008) [40]	Endoscopic endonasal	GTR 4, STR 3	7	23.4	NR	Improved 2, Unchanged 1, Worsened 0	Mean 36.2	No
Fatemi et al. (2009) [29]	Endoscopic endonasal	GTR 3, NTR/STR 15	18	40 (18 – 62)	NR	Improved 11, Unchanged 1, Worsened 0, No impairment preop/postop 6	Mean 20	0% (among 16 Pts)
Campbell et al. (2010) [27]	Endoscopic endonasal	GTR 4, NTR 9	14	45 (18 – 65)	NR	Improved 12, Unchanged 1, Worsened 1	NR	NR
Jane et al. (2010) [35]	Endoscopic endonasal	Radical 6, GTR 5, STR 1	12	50.77 (29 – 76)	Mixed 8, Cystic 3, Solid 1	Improved 7, Unchanged 5, Worsened 0	Mean 13.3	NR
Coppens & Couldwell (2010) [44]	Endoscopic endonasal	STR	1	26	NR	Improved	18	No
Garcia-Navarro et al. (2011) [46]	Endoscopic endonasal	GTR	2	NR	NR	NR	NR	NR
Leng et al. (2012) [49]	Endoscopic endonasal	GTR 21	24	43.6 (5 – 82)	Cystic 10, Solid & cystic 14	Improved 10, Unchanged 3, Worsened 1	Mean 32.9	26%
Koutourousiou et al. (2013) [48]	Endoscopic endonasal	GTR 46, STR 14, PR 4	64 (Primary 47 – Recurrent 17)	40 (4 – 82)	NR	Improved 38, Unchanged 5, Worsened 1	Mean 38	34%
Cavallo et al. (2014) [43]	Endoscopic endonasal	GTR 71, STR 26, PR 6	103	42.5 (3 – 83)	NR	Improved 63, Unchanged 14, Worsened 2	Mean 48	22%.
Gu et al. (2015) [47]	Endoscopic endonasal	GTR	3	36.3	Solid	Improved 3, Unchanged 0, Worsened 0	Mean 35.6	33%
Prabhu et al. (2015) [50]	Endoscopic endonasal	GTR	1	79	Cystic	Improved	NR	NR
Abou-Al-Shaar et al. (2016) [41]	Endoscopic endonasal	GTR	1	22	Cystic & solid	NR	12	No
Bal et al. (2016) [42]	Endoscopic endonasal	GTR 20	25 (Primary 15 – Recurrent 10)	5 – 68	NR	Improved 15, Unchanged 10, Worsened 0	Mean 54.7	NR
Fomichev et al. (2016) [45]	Endoscopic endonasal	GTR 98	136	49.3 (13-73)	NR	Improved + Unchanged 121, Worsened 15	Mean 42	20%
Mangussi-Gomes et al. (2018) [54]	Endoscopic endonasal	NR	1	72	Cystic & solid	NR	NR	NR
Locatelli et al. (2018) [53]	Endoscopic endonasal	NR	1	43	Cystic & solid	Improved	12	No
Vitaz et al. (2001) [59]	Ommaya reservoir	NR	2	9	Cystic	NR	NR	NR
Nicolato et al. (2004) [56]	Ommaya reservoir endoscopy + bleomycin	GTR 3, STR 4, PR 1	8	35.1 (12 – 74)	Cystic	Improved 5, Unchanged 2, Worsened 1	Mean 42.8	12.5%
Park et al. (2011) [15]	Ommaya reservoir endoscopy	STR 13	13	26.0 (4 – 66)	Cystic	Improved 12, Unchanged 0, Worsened 1	Mean 32	54%
Moussa et al. (2013) [55]	Ommaya reservoir + Stereotaxy	NR	52	6 – 42	Cystic	Improved 21, Unchanged 17, Worsened 0	Mean 54	27%
Rahmathulla & Barnett (2013) [51]	Ommaya reservoir + Stereotaxy	STR 4	4	58 (31 – 78)	Cystic	Improved + Unchanged 4	Mean 55	No
Srikandarajah et al. (2014) [57]	Ommaya reservoir	NR	6	NR	Cystic	NR	NR	33%
Takano et al. (2015) [17]	Ommaya reservoir endoscopy	NR	9	56.7 (35 –88)	Cystic	Improved 9	Mean 73	11%
Vakharia et al. (2017) [58]	Ommaya reservoir	NR	1	75	Cystic	NR	NR	NR
Zhu et al. (2017) [60]	Ommaya reservoir + Microsurgery	GTR 8, STR 3	11	7.36	Cystic	Improved + Unchanged 11	Mean 18.6	No
Lauretti et al. (2018) [2]	Ommaya reservoir endoscopy	NR	8	43 (32 – 52)	Cystic & mixed	Improved 8	Mean 56	12.5%
Frio et al. (2019) [52]	Ommaya reservoir endoscopy (8 Pts) + Stereotaxy (3 Pts)	NR	11	49.5 (18 – 77)	Cystic	Improved 5, Unchanged NR, Worsen NR	Mean 41.4	27.3%

The NTVA is recognized as a safe, efficacious, and minimally invasive procedure for intra- and paraventricular craniopharyngiomas, especially for cystic, large, and extensive lesions [2-3]. Our literature review results, as well as our experience exemplified by the case report described above, further support this notion (Table 1, Table 3). Intraoperative fenestration of the cyst wall usually drains the dense liquid content, typically described as ‘engine oil’ in color and texture [2]; spillage of the cyst contents into spaces containing cerebrospinal fluid (CSF) may lead to chemical meningitis and possibly secondary hydrocephalus [32]. Some authors advocate inserting a catheter with an Ommaya reservoir to allow for intermittent drainage, combined with radiation therapy [51]; long-term catheter placement, however, is associated with risks of infection, catheter displacement, content re-accumulation due to cyst septations, and pain with Ommaya reservoir tapping. Remarkably, none of the NTVA studies summarized in Table 1 reported the occurrence of chemical meningitis or delayed hydrocephalus secondary to the communication of CSF spaces with the cyst.

**Table 3:** Comparison of recurrence rates among different surgical modalities N, number of patients

Surgical Approach/Procedure	N	Recurrence rate
Neuroendoscopic transventricular without an Ommaya reservoir	37	0% to 33%
Endoscopy endonasal	540	0% to 34%
Microsurgical	785	0% to 62%
Ommaya reservoir insertion	125	0% to 54%

The low complication and recurrence rates (Table 1 and Table 3) following NTVA, as well as the endoscopic endonasal approach, compared to microsurgery and Ommaya reservoir insertion, support the validity of these minimally invasive techniques as credible and potentially better alternatives in the management of CC or cystic-dominant craniopharyngiomas. This conclusion is limited by the small number of reported NTVA cases and the heterogeneity of the published studies.

## Conclusions

The NTVA is efficient, reliable, and safe for managing CC and cystic-dominant craniopharyngiomas, with potentially lower recurrence and complication rates compared to microsurgery and Ommaya reservoir insertion. Future randomized clinical studies comparing the various treatment modalities of CC are needed to solidify these conclusions.

## References

[1] Gangemi M, Seneca V, Mariniello G, Colella G, Magro F (2009). Combined endoscopic and microsurgical removal of a giant cystic craniopharyngioma in a six-year-old boy. Clin Neurol Neurosurg.

[2] Lauretti L, Legninda Sop FY, Pallini R, Fernandez E, D'Alessandris QG (2018). Neuroendoscopic treatment of cystic craniopharyngiomas: a case series with systematic review of the literature. World Neurosurg.

[3] Nakahara Y, Koga H, Maeda K, Takagi M, Tabuchi K (2004). Neuroendoscopic transventricular surgery for suprasellar cystic mass lesions such as cystic craniopharyngioma and Rathke cleft cyst. Neurol Med Chir (Tokyo).

[4] Pettorini BL, Tamburrini G, Massimi L, Caldarelli M, Di Rocco C (2009). Endoscopic transventricular positioning of intracystic catheter for treatment of craniopharyngioma. Technical note. J Neurosurg Pediatr.

[5] Hellwig D, Bauer BL, List-Hellwig E (1995). Stereotactic endoscopic interventions in cystic brain lesions. Acta Neurochir Suppl.

[6] Nakamizo A, Inamura T, Nishio S, Inoha S, Ishibashi H, Fukui M (2001). Neuroendoscopic treatment of cystic craniopharyngioma in the third ventricle. Minim Invasive Neurosurg.

[7] Joki T, Oi S, Babapour B (2002). Neuroendoscopic placement of Ommaya reservoir into a cystic craniopharyngioma. Childs Nerv Syst.

[8] Delitala A, Brunori A, Chiappetta F (2004). Purely neuroendoscopic transventricular management of cystic craniopharyngiomas. Childs Nerv Syst.

[9] Tirakotai W, Schulte DM, Bauer BL, Bertalanffy H, Hellwig D (2004). Neuroendoscopic surgery of intracranial cysts in adults. Childs Nerv Syst.

[10] Kamikawa S, Inui A (2005). Pediatric malignancies. Case 3. Craniopharyngioma in a 4-year-old girl: neuroendoscopic diagnosis and treatment. J Clin Oncol.

[11] Berlis A, Vesper J, Ostertag C (2006). Stent placement for intracranial cysts by combined stereotactic/endoscopic surgery. Neurosurgery.

[12] Cinalli G, Spennato P, Cianciulli E, Fiorillo A, Di Maio S, Maggi G (2006). The role of transventricular neuroendoscopy in the management of craniopharyngiomas: three patient reports and review of the literature. J Pediatr Endocrinol Metab.

[13] Fujimoto Y, Fujimoto Y, Kato A, Yoshimine T (2007). Neuroendoscopic palliation for large cystic craniopharyngioma in an elderly patient. Br J Neurosurg.

[14] Cappabianca P, Cinalli G, Gangemi M (2008). Application of neuroendoscopy to intraventricular lesions. Neurosurgery.

[15] Park YS, Chang JH, Park YG, Kim DS (2011). Recurrence rates after neuroendoscopic fenestration and Gamma Knife surgery in comparison with subtotal resection and Gamma Knife surgery for the treatment of cystic craniopharyngiomas. J Neurosurg.

[16] Mohanty A, Thompson BJ, Patterson J (2013). Initial experience with endoscopic side cutting aspiration system in pure neuroendoscopic excision of large intraventricular tumors. World Neurosurg.

[17] Takano S, Akutsu H, Mizumoto M, Yamamoto T, Tsuboi K, Matsumura A (2015). Neuroendoscopy followed by radiotherapy in cystic craniopharyngiomas—a long-term follow-up. World Neurosurg.

[18] Shukla D (2015). Transcortical transventricular endoscopic approach and Ommaya reservoir placement for cystic craniopharyngioma. Pediatr Neurosurg.

[19] Moore RJ, Scherer A, Fulkerson DH (2017). Frontal burr hole approach for neuroendoscopic resection of craniopharyngioma with the NICO Myriad device: report of two cases. Childs Nerv Syst.

[20] Yaşargil M (1996). Microneurosurgery. Microsurgery of CNS Tumors. https://www.thieme.com/books-main/neurosurgery/product/2598-microneurosurgery-volume-ivb.

[21] Fahlbusch R, Honegger J, Paulus W, Huk W, Buchfelder M (1999). Surgical treatment of craniopharyngiomas: experience with 168 patients. J Neurosurg.

[22] Im SH, Wang KC, Kim SK, Chung YN, Kim HS, Lee CH, Cho BK (2003). Transsphenoidal microsurgery for pediatric craniopharyngioma: special considerations regarding indications and method. Pediatr Neurosurg.

[23] Karavitaki N, Brufani C, Warner JT (2005). Craniopharyngiomas in children and adults: systematic analysis of 121 cases with long-term follow-up. Clin Endocrinol (Oxf).

[24] Van Effenterre R, Boch AL (2002). Craniopharyngioma in adults and children: a study of 122 surgical cases. J Neurosurg.

[25] Abe T, Ludecke DK (1997). Recent results of primary transnasal surgery for infradiaphragmatic craniopharyngioma. Neurosurg Focus.

[26] Buhl R, Nabavi A, Fritsch M, Mehdorn HM (2001). Nasopharyngeal extension of a craniopharyngioma in a 4 year old girl. Acta Neurochir (Wien).

[27] Campbell PG, McGettigan B, Luginbuhl A, Yadla S, Rosen M, Evans JJ (2010). Endocrinological and ophthalmological consequences of an initial endonasal endoscopic approach for resection of craniopharyngiomas. Neurosurg Focus.

[28] Chakrabarti I, Amar AP, Couldwell W, Weiss MH (2005). Long-term neurological, visual, and endocrine outcomes following transnasal resection of craniopharyngioma. J Neurosurg.

[29] Fatemi N, Dusick JR, de Paiva Neto MA, Malkasian D, Kelly DF (2009). Endonasal versus supraorbital keyhole removal of craniopharyngiomas and tuberculum sellae meningiomas. Neurosurgery.

[30] Feng SY, Zhang YY, Yu XG, Chen XL, Zhou T, Bu B, Jiang JL (2018). Microsurgical treatment of craniopharyngioma. Experiences on 183 consecutive patients. Medicine (Baltimore).

[31] Filis AK, Moon K, Cohen AR (2009). Synchronous ventriculoscopic and microsurgical resection of complex craniopharyngiomas. Pediatr Neurosurg.

[32] Fujimoto Y, Matsushita H, Velasco O, Rosemberg S, Plese JP, Marino R Jr (2002). Craniopharyngioma involving the infrasellar region. A case report and review of the literature. Pediatr Neurosurg.

[33] Gardner PA, Kassam AB, Snyderman CH, Carrau RL, Mintz AH, Grahovac S, Stefko S (2008). Outcomes following endoscopic, expanded endonasal resection of suprasellar craniopharyngiomas: a case series. J Neurosurg.

[34] Ichikawa T, Otani Y, Ishida J, Fujii K, Kurozumi K, Ono S, Date I (2016). Hybrid microscopic-endoscopic surgery for craniopharyngioma in neurosurgical suite: technical notes. World Neurosurg.

[35] Jane JA Jr, Kiehna E, Payne SC, Early SV, Laws ER Jr (2010). Early outcomes of endoscopic transsphenoidal surgery for adult craniopharyngiomas. Neurosurg Focus.

[36] Locatelli D, Levi D, Rampa F, Pezzotta S, Castelnuovo P (2004). Endoscopic approach for the treatment of relapses in cystic craniopharyngiomas. Childs Nerv Syst.

[37] Rudnick EF, DiNardo LJ (2006). Image-guided endoscopic endonasal resection of a recurrent craniopharyngioma. Am J Otolaryngol.

[38] Schubert T, Trippel M, Tacke U (2009). Neurosurgical treatment strategies in childhood craniopharyngiomas: is less more?. Childs Nerv Syst.

[39] Shibata T, Tanikawa M, Sakata T, Mase M (2018). Urgent optic nerve decompression via an endoscopic endonasal transsphenoidal approach for craniopharyngioma in a 12-month-old infant: a case report. Pediatr Neurosurg.

[40] Stamm AC, Vellutini E, Harvey RJ, Nogeira JF Jr, Herman DR (2008). Endoscopic transnasal craniotomy and the resection of craniopharyngioma. Laryngoscope.

[41] Abou-Al-Shaar H, Blitz AM, Rodriguez FJ, Ishii M, Gallia GL (2016). Expanded endonasal endoscopic approach for resection of an infrasellar craniopharyngioma. World Neurosurg.

[42] Bal E, Öge K, Berker M (2016). Endoscopic endonasal transsphenoidal surgery, a reliable method for treating primary and recurrent/residual craniopharyngiomas: nine years of experience. World Neurosurg.

[43] Cavallo LM, Frank G, Cappabianca P (2014). The endoscopic endonasal approach for the management of craniopharyngiomas: a series of 103 patients. J Neurosurg.

[44] Coppens JR, Couldwell WT (2010). Staged use of the transsphenoidal approach to resect superior third ventricular craniopharyngiomas. Minim Invasive Neurosurg.

[45] Fomichev D, Kalinin P, Kutin M, Sharipov O (2016). Extended transsphenoidal endoscopic endonasal surgery of suprasellar craniopharyngiomas. World Neurosurg.

[46] Garcia-Navarro V, Lancman G, Guerrero-Maldonado A, Anand VK, Schwartz TH (2011). Use of a side-cutting aspiration device for resection of tumors during endoscopic endonasal approaches. Neurosurg Focus.

[47] Gu Y, Zhang X, Hu F, Yu Y, Xie T, Sun C, Li W (2015). Suprachiasmatic translamina terminalis corridor used in endoscopic endonasal approach for resecting third ventricular craniopharyngioma. J Neurosurg.

[48] Koutourousiou M, Gardner PA, Fernandez-Miranda JC, Tyler-Kabara EC, Wang EW, Snyderman CH (2013). Endoscopic endonasal surgery for craniopharyngiomas: surgical outcome in 64 patients. J Neurosurg.

[49] Leng LZ, Greenfield JP, Souweidane MM, Anand VK, Schwartz TH (2012). Endoscopic, endonasal resection of craniopharyngiomas: analysis of outcome including extent of resection, cerebrospinal fluid leak, return to preoperative productivity, and body mass index. Neurosurgery.

[50] Prabhu V, Anand VK, Schwartz TH (2015). Preservation of pituitary function after endonasal craniopharyngioma surgery: case report and review of the literature. Cureus.

[51] Rahmathulla G, Barnett GH (2013). Minimally invasive management of adult craniopharyngiomas: an analysis of our series and review of literature. Surg Neurol Int.

[52] Frio F, Solari D, Cavallo LM, Cappabianca P, Raverot G, Jouanneau E (2019). Ommaya reservoir system for the treatment of cystic craniopharyngiomas: surgical results in a series of 11 adult patients and review of the literature. World Neurosurg.

[53] Locatelli D, Pozzi F, Agresta G, Padovan S, Karligkiotis A, Castelnuovo P (2018). Extended endoscopic endonasal approach for suprasellar craniopharyngioma. J Neurol Surg B Skull Base.

[54] Mangussi-Gomes J, Vellutini EA, Truong HQ, Pahl FH, Stamm AC (2018). Endoscopic endonasal transplanum transtuberculum approach for the resection of a large suprasellar craniopharyngioma. J Neurol Surg B Skull Base.

[55] Moussa AH, Kerasha AA, Mahmoud ME (2013). Surprising outcome of ommaya reservoir in treating cystic craniopharyngioma: a retrospective study. Br J Neurosurg.

[56] Nicolato A, Foroni R, Rosta L, Gerosa M, Bricolo A (2004). Multimodality stereotactic approach to the treatment of cystic craniopharyngiomas. Minim Invasive Neurosurg.

[57] Srikandarajah N, Patel A, Lee MK, Brodbelt A (2014). Indications for intracranial reservoirs: a six-year study. Br J Neurosurg.

[58] Vakharia K, Siasios ID, Dorsch AB, Leonardo J (2017). Spontaneous intraventricular rupture of a craniopharyngioma cyst: a case report. Int J Crit Illn Inj Sci.

[59] Vitaz TW, Hushek S, Shields CB, Moriarty T (2001). Changes in cyst volume following intraoperative MRI-guided Ommaya reservoir placement for cystic craniopharyngioma. Pediatr Neurosurg.

[60] Zhu W, Li X, He J, Sun T, Li C, Gong J (2017). A reformed surgical treatment modality for children with giant cystic craniopharyngioma. Childs Nerv Syst.

